# Isotalatizidine, a C_19_-diterpenoid alkaloid, attenuates chronic neuropathic pain through stimulating ERK/CREB signaling pathway-mediated microglial dynorphin A expression

**DOI:** 10.1186/s12974-019-1696-9

**Published:** 2020-01-10

**Authors:** Shuai Shao, Huan Xia, Min Hu, Chengjuan Chen, Junmin Fu, Gaona Shi, Qinglan Guo, Yu Zhou, Wenjie Wang, Jiangong Shi, Tiantai Zhang

**Affiliations:** State Key Laboratory of Bioactive Substances and Functions of Natural Medicines, Institute of Materia Medica, Chinese Academy of Medical Sciences & Peking Union Medical College, Beijing, China

**Keywords:** Isotalatizidine, Neuropathic pain, Microglia, ERK1/2 MAPK, CREB, Dynorphin A

## Abstract

**Background:**

Isotalatizidine is a representative C_19_-diterpenoid alkaloid extracted from the lateral roots of *Aconitum carmichaelii*, which has been widely used to treat various diseases on account of its analgesic, anti-inflammatory, anti-rheumatic, and immunosuppressive properties. The aim of this study was to evaluate the analgesic effect of isotalatizidine and its underlying mechanisms against neuropathic pain.

**Methods:**

A chronic constrictive injury (CCI)-induced model of neuropathic pain was established in mice, and the limb withdrawal was evaluated by the Von Frey filament test following isotalatizidine or placebo administration. The signaling pathways in primary or immortalized microglia cells treated with isotalatizidine were analyzed by Western blotting and immunofluorescence.

**Results:**

Intrathecal injection of isotalatizidine attenuated the CCI-induced mechanical allodynia in a dose-dependent manner. At the molecular level, isotalatizidine selectively increased the phosphorylation of p38 and ERK1/2, in addition to activating the transcription factor CREB and increasing dynorphin A production in cultured primary microglia. However, the downstream effects of isotalatizidine were abrogated by the selective ERK1/2 inhibitor U0126-EtOH or CREB inhibitor of KG-501, but not by the p38 inhibitor SB203580. The results also were confirmed in in vivo experiments.

**Conclusion:**

Taken together, isotalatizidine specifically activates the ERK1/2 pathway and subsequently CREB, which triggers dynorphin A release in the microglia, eventually leading to its anti-nociceptive action.

## Background

**Fig. 1:**
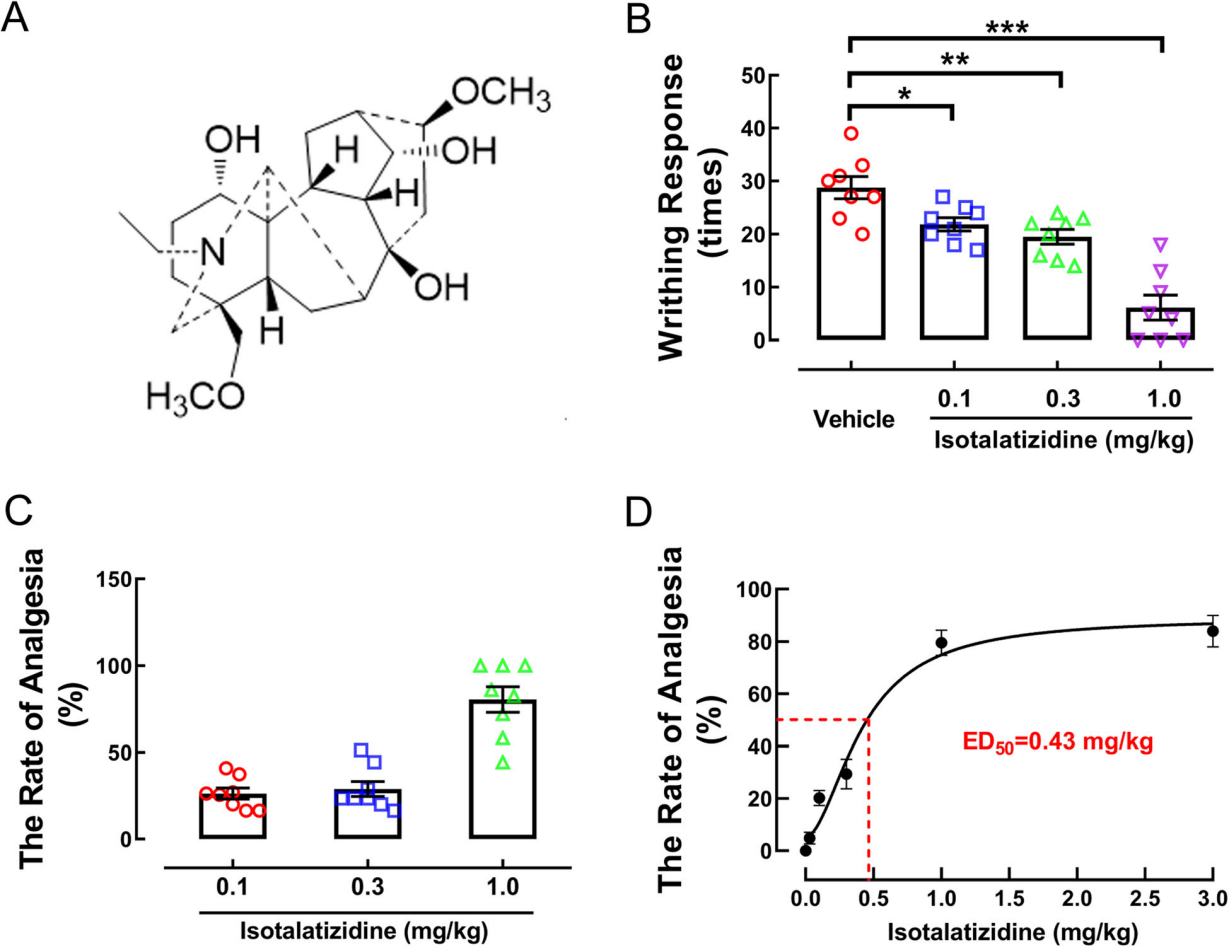
The effect of isotalatizidine on acetic acid-induced somatic pain in mice. (**a**) The chemical structure of isotalatizidine. Isotalatizidine significantly decreased the writhing times (**b**) and increased the rate of analgesia (**c**) in acetic acid-induced mice. The ED_50_ of analgesic efficiency of isotalatizidine was 0.43 mg/kg (**d**). Data are expressed as mean ± SEM (*n* = 8 mice in each group). ^*^P < 0.05, ^**^P < 0.01, ^***^P < 0.001 vs. vehicle group

Neuropathic pain is caused by an injury or disease in the somatosensory nervous system, including the central nerves, spinal cord, posterior root of the spinal cord, and peripheral nerves. It is highly prevalent and has an immense impact on the quality of life of patients [1]. Opiate agents and non-steroidal anti-inflammatory drugs are classically used to control pain, but are associated with gastrointestinal disturbances and possible risk of dependence [2]. Recently, the focus has greatly shifted to natural plant-derived antinociceptive agents with high efficacy and safety. The main and lateral roots of *Aconitum carmichaelii* Debx (Ranunculaceae), known as “Wutou” and “Fuzi”, respectively, in Chinese, are widely used in traditional Chinese medicine as analgesic, anti-inflammatory, anti-rheumatic, and immunosuppressive agents [3–5]. More than a hundred chemicals have been isolated from the different parts of this herb [6], of which the diterpenoid alkaloids are the main pharmacologically active constituents. Various *A. carmichaelii* compounds were screened in an acetic acid-induced murine pain model, and the C_19_-diterpenoid alkaloid isotalatizidine (Fig. 1a) displayed a significant analgesic effect. However, no scientific reports have been published so far on the mechanisms underlying its analgesic properties.

Increasing evidence shows that the progression of neuropathic pain is closely related to microglial cells in the spinal cord [7, 8]. Microglia account for only 5–12% of the cells in the central nervous system (CNS) but play a crucial role in sensing internal stimuli, transmitting excitatory signals and regulating physiological functions [9]. In addition, the microglial cells are also the resident macrophages of the CNS tissues and therefore form part of the local innate immune response [10]. Following harmful stimuli or nerve injuries, various immune cells are rapidly mobilized and activated and release chemokines and cytokines that induce peripheral sensitization and microglial activation in the peripheral and central nervous system [11, 12]. However, persistent activation of the microglial cells can elevate neuronal excitability and maintain the transmission of pain signals to the spinal dorsal horn neurons [12, 13]. Indeed, the basis of neuropathic pain is the production of pro-inflammatory and pro-nociceptive mediators such as interleukins (IL-1β, IL-6, IL-12, IL-15, IL-18) [14], IFN-γ, TNF-α [15], and chemokines (CCL2, CCL3, CCL4, CCL5, CCL7) [16, 17] by the constitutively active microglia cells [12, 18, 19]. The mitogen-activated protein kinase (MAPKs) family of proteins, including extracellular signal-regulated kinase (ERK), p38, and c-Jun N-terminal kinase (JNK), plays a crucial role in the signaling pathways mediating microglial activation and nociceptive responses, which eventually lead to neuropathic pain [20, 21]. Therefore, targeting the microglial signaling pathways can help understand the complex mechanisms underlying neuropathic pain and provide novel insights into drug discovery. Dynorphin A, an endogenous neurotransmitter expressed by neurons, microglia, and astrocytes, mediates neuropathic pain via its distinct κ-opioid receptor [22–24]. The production of dynorphin A involves multiple transcription factors [25, 26], including the cAMP response element-binding protein (CREB) which induces the transcription of the dynorphin A precursor prodynorphin [27]. As an upstream regulator of CREB, phosphorylated MAPK is crucial to prodynorphin expression and dynorphin A release in microglia. Although dynorphin A is elevated during neuropathic pain, it is not clear whether it is pro- or anti-nociceptive.

The aim of the present study was to evaluate the anti-nociceptive effects of isotalatizidine and explore the relevant signaling pathways in microglial cells, in order to determine its possible mechanism against neuropathic pain.

## Materials and methods

### Drugs and reagents

Isotalatizidine was extracted and purified by the Institute of Materia Medica, Chinese Academy of Medical Sciences and Peking Union Medical College, and the purity was validated as > 95% using high-performance liquid chromatography. For the experiments, it was dissolved in dimethyl sulfoxide (DMSO) and subsequently diluted in sterile saline (0.9%). SB203580 and U0126-EtOH were all purchased from TargetMol (Shanghai, China). KG-501 was purchased from MedChemExpress (Shanghai, China) and dissolved in DMSO, and diluted with DMEM, DMEM/F-12, or saline before use. The cell culture reagents were purchased from the Invitrogen Corporation (Thermo Fisher Scientific, Carlsbad, CA, USA). Anti-dynorphin A antibody was purchased from Abcam (Cambridge, UK), and the Alexa 546-conjugated goat anti-rabbit and Alexa 488-conjugated goat anti-mouse secondary antibodies from the Life Technology (Thermo Fisher Scientific, Carlsbad, CA, USA). The remaining antibodies were purchased from the Cell Signaling Technology (Beverly, MA, USA). The goat serum was purchased from the Beyotime Biotechnology (Shanghai, China), and triton X-100 from the Sigma Aldrich.

### Experimental animals

The ICR mice (female, weighing18–20 g) and C57BL/6 mice (females, 18–20 g) were obtained from the Beijing Huafukang Experimental Animal Institute (Beijing, China). The adult mice (5–6 per cage) were housed at room temperature (22 ± 2 °C) in specific pathogen-free conditions under a 12/12-h reversed light-dark cycle, with food and water provided ad libitum. The mice were acclimatized for 3–4 days before the experiments and randomly divided into the different groups. Animal studies were conducted following the protocols approved by the Experimental Animal Welfare and Ethics Committee of the Institute of Materia Medica, Chinese Academy of Medical Sciences. Animal studies are reported in compliance with the ARRIVE guidelines [28]. The experimental designs were based on the rule of the replacement, refinement, and reduction to reduce suffering of the animals and use the minimum number of animals.

### Establishment of somatic or neuropathic pain model and treatment

#### Acetic acid-induced abdominal writhing test

Acetic acid-induced mouse somatic pain model was used to evaluate the analgesic effect of isotalatizidine. ICR mice were pre-treated with isotalatizidine (0.1, 0.3, or 1.0 mg/kg) or vehicle (normal saline, 1 ml/kg) by a single intraperitoneal injection. Thirty minutes later, obtained 1.0% acetic acid solution (10 ml/kg) was injected intraperitoneally. The times of writhes and stretching were counted over a period of 15 min after acetic acid injection.

#### Chronic constrictive injury (CCI)-induced neuropathic pain model test

Chronic neuropathic pain following peripheral nerve injury was simulated by chronic constrictive injury (CCI) of the unilateral sciatic nerve as described previously [29]. Briefly, the C57BL/6 mice were anesthetized with isoflurane and randomly divided into the sham-operated, untreated CCI model, and isotalatizidine-treated (0.1, 0.3, and 1 mg/kg) groups (*n* = 6 each). The left sciatic nerve trunk was exposed by blunt dissection at mid-thigh level, and 4 ligatures (4–0 chromic catgut) were tied loosely around the nerve with 1 mm spacing. The control mice were subjected to sham surgery wherein the sciatic nerve was only exposed but not ligated.

### Intrathecal injection

On the eighth day after surgery, the mice were given a single intrathecal injection of the suitable isotalatizidine dose or saline as described previously by Hylden and Wilcox [30] with slight modifications. Briefly, the mice were anesthetized with isoflurane (4% for induction and 1% for maintenance), and a 100 μl micro-injector was inserted from the intervertebral space between the L5 and L6 spinal cord into the spinal subarachnoid space. After confirming proper intrathecal injection by tail flicking, 100 μl normal saline or drug was microinjected followed by a 100-μl normal saline flush.

### Behavioral analysis

Acetic acid-induced writhing was evaluated by counting the number of writhes and stretches over 15 min after its injection. The sensitivity of mechanical nociception in the CCI model was measured in terms of the withdrawal threshold of the ipsilateral and contralateral limbs by the Von Frey test (Von Frey filaments, IITC Life Science Inc, California, USA) after 30 min, 1 h, 2 h, and 4 h of intrathecal injection. The animals were acclimatized in boxes set on an elevated metal mesh floor for at least 30 min. A series of monofilaments of different pressure values were pressed vertically on the sole of the hind paws with an increasing force till the animal withdrew the hindlimb. The procedure was repeated 5 times, and the average threshold value was calculated. All behavioral analyses were performed by an investigator blinded to the experimental grouping.

### BV-2 and primary microglial cell culture

BV-2 cells were cultured in Dulbecco’s modified Eagle’s medium (DMEM) supplemented with 10% fetal bovine serum (FBS), 100 U/ml penicillin, 100 μg/m streptomycin, and 5.5 mM glucose at 37 °C under 5% CO_2_ and 95% humidity. To isolate primary microglia cells, spinal cords were removed from 1-day-old Wistar rats (Beijing Huafukang Experimental Animal Institute) and minced in ice-cold D-Hank’s medium containing penicillin (100 U/ml) and streptomycin (100 μg/ml). After digesting with 0.125% trypsin, the dissociated cells were suspended in equal volume of complete DMEM/F12 medium (supplemented with 10% FBS, 100 U/ml penicillin and 100 μg/ml streptomycin) to stop the reaction. The cell suspension was then filtered through a 200-μm mesh screen to remove tissue debris and centrifuged at 1000 rpm for 5 min. The pellet was re-suspended in DMEM/F12 medium and plated onto poly-l-lysine pre-coated (100 μg/ml) 75-cm^2^ tissue culture flasks. The primary microglial cells were cultured at 37 °C under 5% CO_2_ for 10 days and harvested by shaking the flasks at 180 rpm for 5 h. Multiple aliquots were then re-plated, and unattached cells were removed by washing with serum-free DMEM. The final harvested microglial cells were identified by IBA-1 immunoreactivity and exhibited > 95% purity.

### RNA isolation and qRT-PCR

Total RNA was extracted from spinal dorsal lumbar enlargements and BV-2 in TRIzol (Invitrogen, Carlsbad, CA, USA) on ice and then reverse transcribed into cDNA and subjected to qPCR. QPCR was performed with Roche LightCycler 480 PCR Detection System (Roche, Switzerland). The fold change was calculated using the 2^-ΔΔCt^ method after normalization to *GAPDH*. The primer sequences are as follows: *GAPDH* (5′-ATC CCA TCA CCA TCT TCC AGG AG-3′ and 5′ CCT GCT TCA CCA CCT TCT TGA TG 3′) and *Prodynorphin* (5′-CGG AAC TCC TCT TGG GGT AT-3′ and 5′-CGG AAC TCC TCT TGG GGT AT-3′).

### Protein extraction and Western blotting

BV-2 and primary microglia cells were seeded into 12-well plates at the density of 2 × 10^6^ cells per well. After overnight culture, the cells were pre-treated with 25 μM isotalatizidine for 1 h, harvested, and lysed to extract protein. The spinal dorsal lumbar enlargements were separated from CCI mice after measurement of mechanical withdrawal threshold and then lysed to extract protein. The protein concentration in the cell and tissue lysates were determined by BCA Protein Assay Kit (Thermo Fisher Scientific, Carlsbad, CA, USA), and 20 μg of proteins per sample was separated by 8% sodium dodecyl sulfate-polyacrylamide gel electrophoresis (SDS-PAGE). The bands were transferred to a polyvinylidene fluoride (PVDF) membrane (Millipore Corp., Bedford, MA, USA) that was then blocked with 5% bovine serum albumin (BSA) in tris-based saline-Tween 20 (TBST) at room temperature for 1 h. After incubating overnight with the primary antibodies against p-p38, p-ERK, p-JNK, p-CREB, p38, ERK, JNK, CREB, dynorphin A (all diluted 1:1000), *β*-tubulin, and GAPDH (1:5000 each) at 4 °C, the blots were then incubated with horseradish peroxidase-conjugated goat anti-rabbit and goat anti-mouse secondary antibodies [31]. The membrane was developed using enhanced chemiluminescence reagents (Perkinelmer, USA), and the bands were visualized with Tanon 2000 Imaging system (Beijing, China) and their intensities were quantified using ImageJ Software (NIH, USA).

### Dynorphin A detection

The spinal cord was washed with ice-cold saline and the spinal dorsal lumbar enlargements rapidly dissected and then immediately frozen in liquid nitrogen. Thawed tissue with ice-cold saline was disrupted using a protein homogenizer and centrifugated at 12,000 × *g* for 15 min at 4 °C. Protein concentrations were determined by the use of the BCA Protein Assay Kit (Thermo Fisher Scientific, Carlsbad, CA, USA) with bovine serum albumin as a standard. Dynorphin A immunoassay was performed using a commercial enzyme-linked immunosorbent assay (ELISA) kit (Jiangsu Meimian Co., Nanjing, China) according to the instruction. Standard curves were constructed using known concentrations of dynorphin A by a non-linear regression analysis (Prism, GraphPad Inc, San Diego, CA). The dynorphin A content in the spinal cord extracts was determined from the standard curve done in parallel assays.

### Immunofluorescence

Primary microglia were seeded into a 12-well plate (2 × 10^6^ cells per well) pre-coated with poly-l-lysine (100 μg/ml) and cultured overnight. After treating with isotalatizidine or MAPK inhibitors, the cells were fixed with 4% paraformaldehyde for 30 min at room temperature and permeabilized with 0.2% triton X-100 in phosphate-buffered saline (PBS) containing 10% goat serum for 1 h. The cells were then incubated overnight with anti-dynorphin A (1:200) and anti-IBA-1 (1:200) primary antibodies at 4 °C, washed with PBS, and then incubated with goat anti-rabbit Alexa Fluor 546- and goat anti-mouse Alexa Fluor 488-conjugated secondary antibody (1:200) for 1 h at 37 °C. The nuclei were counterstained with 4, 6-diamidino-2-phenylindole (1 μg/ml, Sigma-Aldrich), washed thrice with PBS, and imaged using Cytation 5 imaging reader (BioTek, VT, USA) [32].

### Statistical analysis

Data were presented as the mean ± SEM, and *p <* 0.05 was considered statistically significant. The 50% mechanical withdrawal threshold (MWT (g)) in the Von Frey test was calculated as 10^[log(*f*·10000) + *k*δ]^/10000 and compared by two-factor analysis of variance (ANOVA) followed by Tukey’s post hoc test using Prism (version 5.01, GraphPad Software Inc., San Diego, CA, USA). The other experiments were analyzed using one-way ANOVA followed by an appropriate post hoc test. The in vitro assays were performed at least thrice.

## Results

### Isotalatizidine attenuates pain hypersensitivity of somatic pain

The analgesic effect of isotalatizidine was tested by the acetic acid-induced writhing test, which showed that this drug significantly reduced the number of writhes compared to the placebo in a dose-dependent manner (Fig. 1b). The analgesic efficiencies were 26.37%, 30.43%, and 76.23% at the doses of 0.1, 0.3, and 1 mg/kg, respectively, (Fig. 1c). Accordingly, the analgesic ED_50_ of isotalatizidine was calculated to be 0.43 mg/kg (Fig. 1d).

### Isotalatizidine treatment alleviated CCI-induced neuropathic pain

**Fig. 2:**
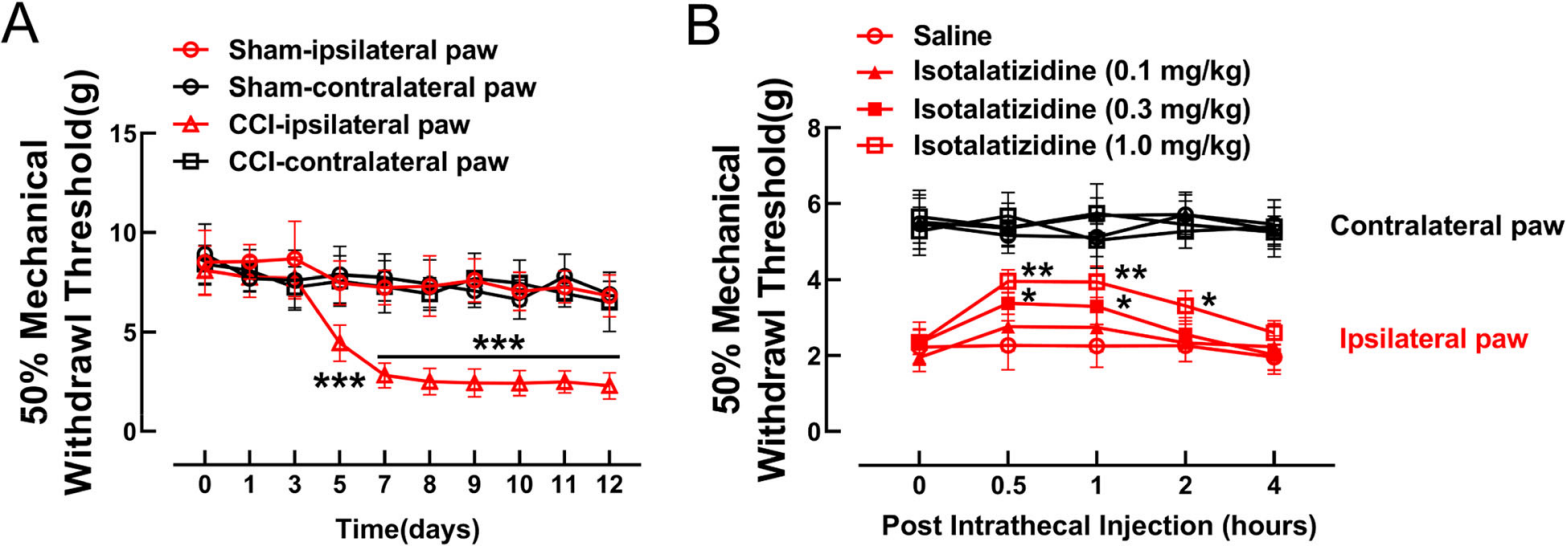
Isotalatizidine treatment improved sciatic nerve ligation-induced chronic neuropathic pain. Neuropathic pain mice were induced by tight ligation of sciatic nerve, the withdrawal thresholds within 12 days after surgery (**a**) and intrathecal injection of vehicle (0.9% normal saline) or isotalatizidine (0.1, 0.3, 1.0 mg/kg) 8 days after surgery (**b**) were measured by Von Frey Monofilament in both contralateral and ipsilateral paws. Data are expressed as means ± SEM (*n* = 6 mice in each group). ^*^P < 0.05, ^**^P < 0.01 vs. saline group, ^***^P < 0.001 vs. sham group

We next determined its effects on mechanical allodynia in a CCI-induced neuropathic pain model by evaluating limb withdrawal. As shown in Fig. 2a, the mechanical withdrawal threshold of the ipsilateral but not the contralateral paw decreased gradually after sciatic nerve ligation and reached a lower level on the eighth day post-surgery. However, isotalatizidine increased this threshold in the ipsilateral paw in a dose-dependent manner compared to the placebo-treated mice, but had no effect on the contralateral paw (Fig. 2b). In addition, no apparent sedative or motor side effects of isotalatizidine were observed during the entire treatment duration.

### Isotalatizidine promoting the phosphorylation of p38 and ERK1/2 in microglial cells but not JNK

**Fig. 3:**
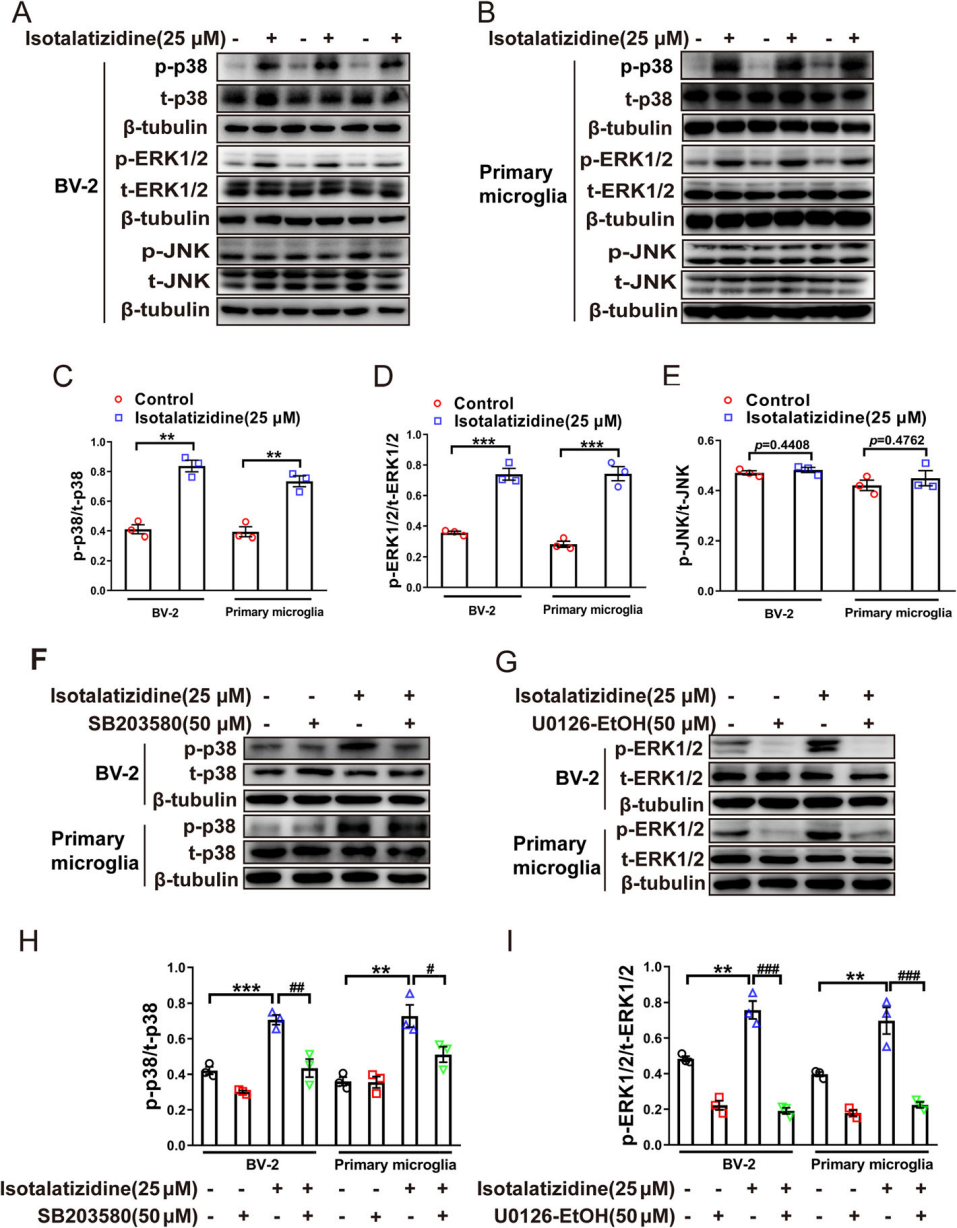
Effects of isotalatizidine on phosphorylation of p38, ERK1/2, and JNK in BV-2 and primary microglial cells. Effects of isotalatizidine on phosphorylation of p38, ERK1/2, and JNK in BV-2 (**a**) and primary microglial cells (**b**). Isotalatizidine (25 μM) obviously promoted the phosphorylation of p38 (**c**) and ERK1/2 (**d**), but not JNK (**e**) in cultured microglial cells. Selective p38 inhibitor SB 203580 (**f**, **h**) and ERK1/2 inhibitor U0126-EtOH (**g**, **i**) significantly blocked the isotalatizidine activated phosphorylation of p38 or ERK1/2, respectively. The data are expressed as mean ± SEM of three independent experiments. ^**^P < 0.01, ^***^P < 0.001 vs. control group; ^#^*P* < 0.05, ^##^*P* < 0.01, ^###^*P* < 0.001 vs*.* isotalatizidine group

MAPK family includes three different signaling cascades of p38, ERK1/2, and JNK, and the phosphorylation of MAPKs plays a critical role in the activation of microglial cells. To investigate the effects of isotalatizidine on MAPK signal activation and selectivity for members of MAPKs, primary microglial and BV-2 cells were applied to test the phosphorylated level of p38, ERK1/2, and JNK under the selective inhibitors by western blotting. The results showed that isotalatizidine treatment at 25 μΜ for 1 h significantly increased the phosphorylation of p38 (*p* < 0.01, Fig. 3a–c) and ERK1/2 (*p* < 0.001, Fig. 3a, b, and d) both in cultured primary microglia and BV-2 cells. However, the same concentration and treatment of iaotalatizidine had no significant change on level of JNK phosphorylation both in cultured primary microglia and BV-2 cells (Fig. 3a, b, and e).

To further explore the causal relationship of MAPK members with isotalatizidine, the selective MAPK subtype inhibitors were applied to determine the responsible subtypes for isotalatizidine. Pre-treatment of the microglial cells with the p38 inhibitor SB203580 (50 μM) and ERK1/2 inhibitor U0126-EtOH (50 μM) significantly abrogated the effects of isotalatizidine (Fig. 3f–i). These results strongly suggested that the analgesic effect of isotalatizidine is likely related to the activation of p38 and the ERK1/2 MAPK signaling pathways in the microglia.

### Isotalatizidine stimulating the CREB activation and dynorphin A release via ERK1/2 pathway in the primary microglial cells

**Fig. 4:**
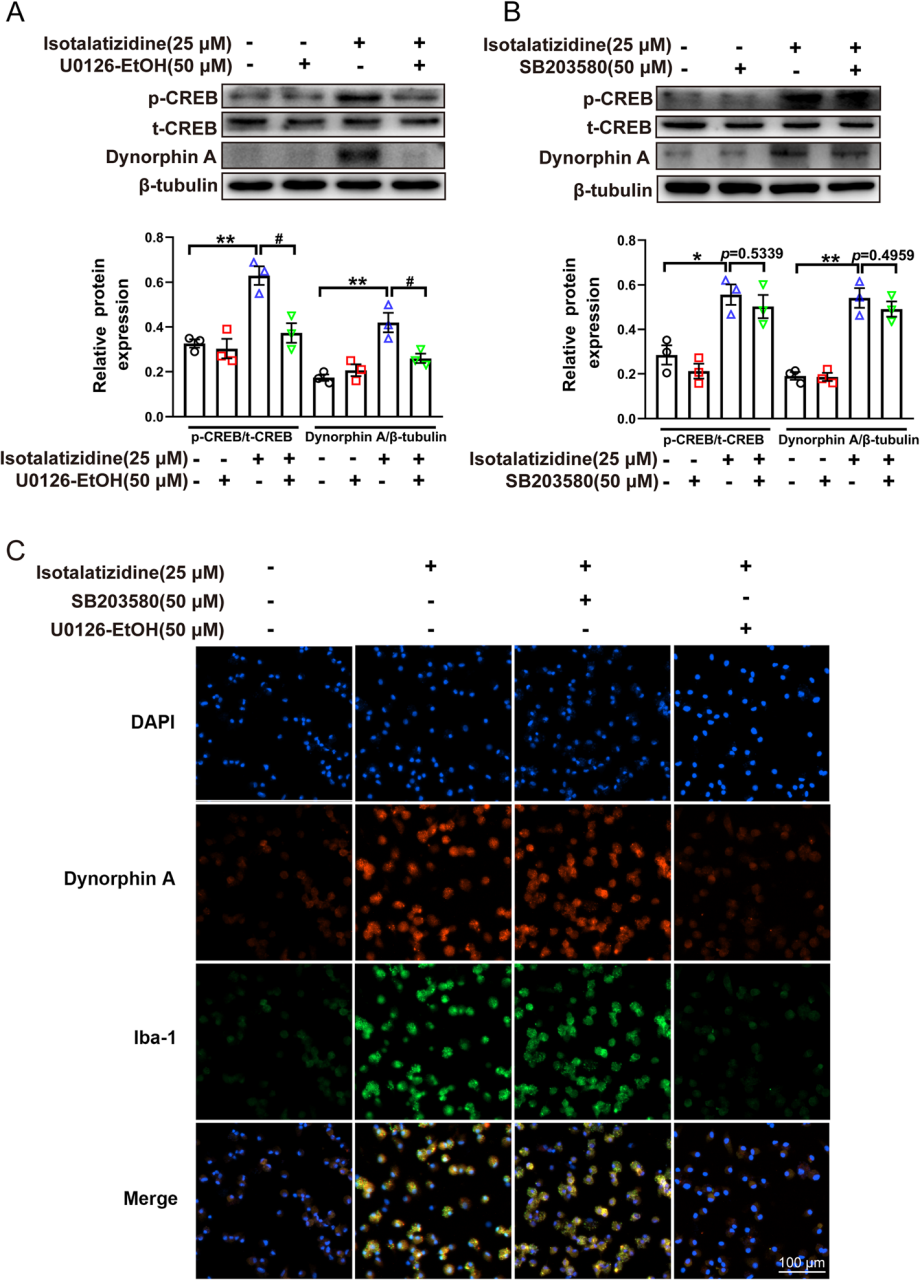
Isotalatizidine stimulating the CREB activation and dynorphin A expression via ERK1/2 pathway in the primary microglial cells. In cultured primary microglial cells, isotalatizidine (25 μM) markedly induced the phosphorylation of CREB and expression of dynorphin A, and the phosphorylated levels of CREB and increased dynorphin A level were markedly prevented by ERK1/2 inhibitor of U0126-EtOH (**a**) but not p38 inhibitor of U0126-EtOH (**b**). Immunofluorescence analysis showed that isotalatizidine-stimulated IBA-1 expression and dynorphin A production also could be blocked by ERK1/2 inhibitor of SB203580 (**c**). The data are expressed as mean ± SEM (*n* = 3). ^*^P < 0.05, ^**^P < 0.01 vs*.* control group; ^#^*P* < 0.05 vs*.* isotalatizidine group

Given the important role of MAPK in CREB activation, we investigated the relationship between phosphorylation of CREB and isotalatizidine in primary microglia. To confirm the correlation between CREB changes with subtype of MAPKs, the selective p38 inhibitor of SB203580 or ERK1/2 inhibitor of U0126-EtOH were used in this assay. As shown in Fig. 4, treatment with isotalatizidine at concentration of 25 μM induced an obvious phosphorylation of CREB in primary microglial cells. However, the phosphorylated levels of CREB elevated by isotalatizidine were markedly prevented by ERK1/2 inhibitor of U0126-EtOH (Fig. 4a, c) but not p38 inhibitor of SB203580 (Fig. 4b, c) in cultured primary microglial cells at 50 μM concentration. The results implied that isotalatizidine-increased phosphorylated level of CREB might be mediated by ERK1/2 MAPK. Next, under the same conditions, the release of dynorphin A was determined by immunofluorescence assay and Western blotting. The results indicated that only ERK1/2 inhibitor of U0126-EtOH obviously reduced the increased level of dynorphin A by isotalatizidine (Fig. 4a, c). However, p38 inhibitor of SB203580 had no effect for the isotalatizidine-induced dynorphin A release (Fig. 4b, c). The above results implied that isotalatizidine-stimulated phosphorylated CREB and dynorphin A release might be mediated by ERK1/2 MAPK.

### Isotalatizidine exerts its analgesic effect by increasing dynorphin A production in activated spinal microglial cells via the ERK1/2-CREB pathway

**Fig. 5:**
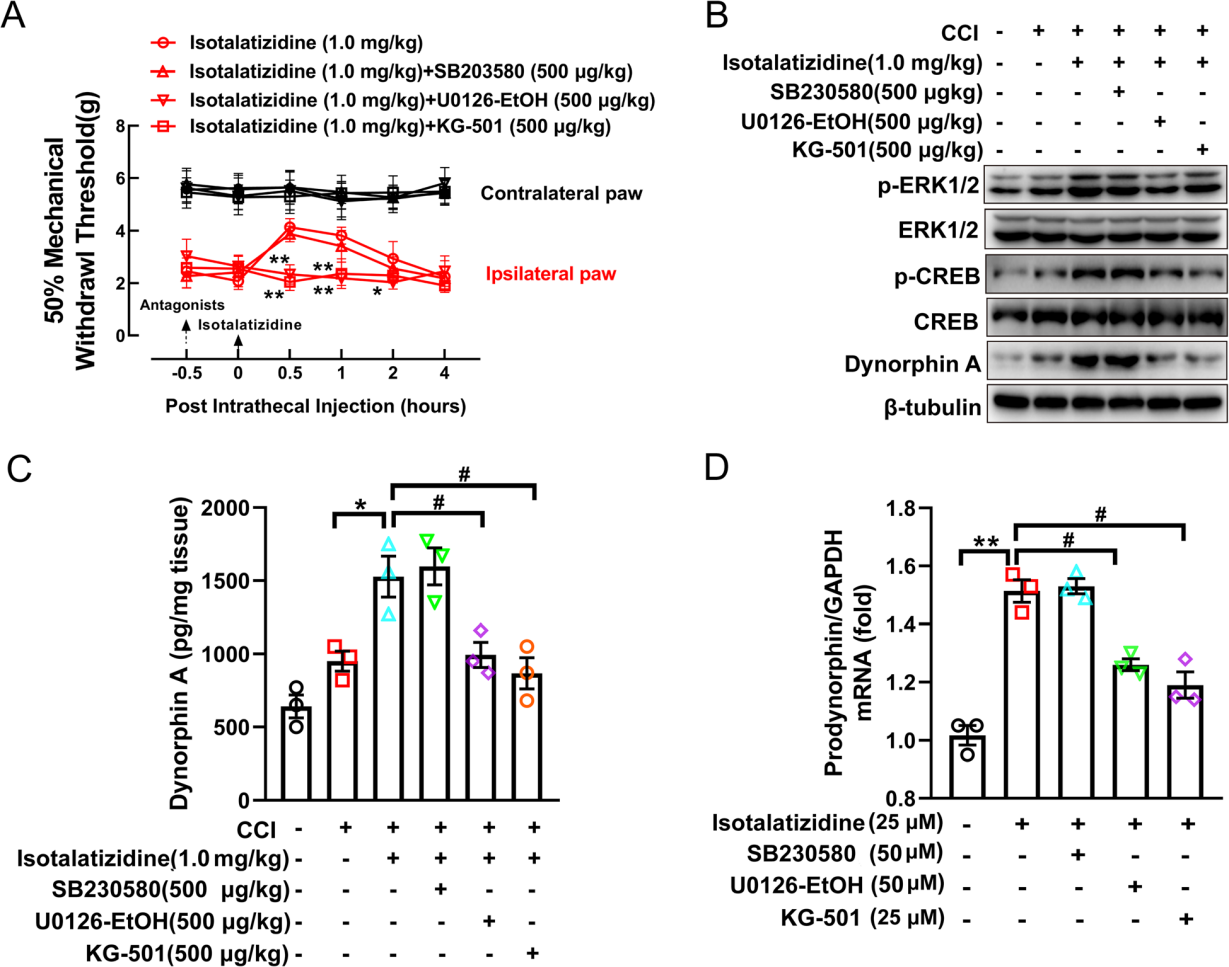
Isotalatizidine exerts analgesic effect by increasing dynorphin A in the spinal cord tissue via the ERK1/2-CREB pathway. Isotalatizidine (1 mg/kg, *n* = 6) induced a time-dependent mechanical antiallodynia of ipsilateral paws in CCI-induced mice, which was completely prevented by ERK inhibitor of U0126-EtOH and CREB inhibitor of KG-501, but not p38 inhibitor of SB203580 (**a**). Phosphorylated level of ERK1/2 and CREB and expression of dynorphin A stimulated by isotalatizidine treatment could be blocked by U0126-EtOH or KG-501 in spinal dorsal tissue, respectively (**b**). Isotalatizidine treatment resulted in the releasing of dynorphin A in spinal cord tissue, which could be suppressed by U0126-EtOH or KG-501, but not SB230580 (**c**). Data are expressed as means ± SEM. ^*^P < 0.05 vs*.* CCI mice; ^#^*P* < 0.05 vs*.* isotalatizidine treated CCI mice. In cultured BV-2 cells, isotalatizidine-induced mRNA expression of prodynorphin also could be inhibited by U0126-EtOH or KG-501, but not SB230580 (**d**). Data are expressed as means ± SEM of three independent experiments. ^**^P < 0.01 vs*.* control group; ^#^*P* < 0.05 vs*.* isotalatizidine group

To explore whether the analgesic effect of isotalatizidine in chronic neuropathic pain mice is mediated by ERK1/2-CREB-dynorphin A axis, CCI mice model was established to measure the relationship between mechanical withdrawal threshold and the activation of ERK1/2-CREB pathway. As shown in Fig. 5, isotalatizidine (1 mg/kg) treatment by intrathecal injection induced a time-dependent mechanical anti-allodynia in the ipsilateral paws, which was completely prevented by the pretreatment of ERK1/2 inhibitor of U0126-EtOH or CREB inhibitor of KG-501, respectively, but not p38 inhibitor of SB203580 (Fig. 5a). Further investigating indicated that isotalatizidine induced a significant phosphorylation of ERK1/2 and CREB and release of dynorphin A in spinal dorsal tissue in CCI mice (Fig. 5b). Our results further displayed that the elevated level of phosphor-ERK1/2 and phosphor-CREB and expression of dynorphin A were all blocked by selective REK1/2 inhibitor of U0126-EtOH. Meanwhile, selective CREB inhibitor of KG-501 also could suppress the phosphorylated level of CREB and expression of dynorphin A induced by isotalatizidine, but not the change of phosphor-ERK1/2. It is known that only secreted dynorphin A binds to opioid receptor inducing the regulatory effect. Therefore, the level of dynorphin A was detected by ELISA in spinal dorsal tissue, the results showed that isotalatizidine significantly increased the secretion of dynorphin A, and this elevation could be blocked by ERK1/2 inhibitor of U0126-EtOH or CREB inhibitor of KG-501, respectively (Fig. 5c). To further conform the relationship between the production of dynorphin A and ERK1/2-CREB signal, the mRNA expression of prodynorphin, precursor of dynorphin A, also was measured in microglia of BV-2. The data indicated that isotalatizidine increased the expression of prodynorphin in BV-2 cell, and these elevated expression of prodynorphin could be prevented by ERK1/2 inhibitor of U0126-EtOH or CREB inhibitor of KG-501, but not the p38 inhibitor of SB230580 (Fig. 5d). Taken together, our data demonstrated that isotalatizidine exerts analgesic effects by releasing dynorphin A which is mediated by activating the ERK1/2-CREB pathway in microglial cells.

## Discussion

Our study shows for the first time that isotalatizidine extracted from *A. carmichaelii* can effectively block the mechanical allodynia in a CCI-induced neuropathic pain model, as well as suppressing writhing in a somatic pain model. Mechanistically, isotalatizidine might induce the phosphorylation of ERK1/2 and CREB, which results in the production of dynorphin A in microglia and exerts analgesic effect.

The lateral root of *A. carmichaelii* Debx has been used for centuries in China, Japan, and Korea to relieve rheumatoid arthritis pain. It has also shown pharmacological effects against cardiovascular diseases, rheumatic fever, joint pain, bronchial asthma, gastroenteritis, collapse, syncope, diarrhea, and edema [33]. Isotalatizidine is a C_19_-diester and monoester diterpenoid alkaloid belonging to the amine alcohol type that is extracted from the lateral roots of *A. carmichaelii* and exhibits low toxicity and potent analgesic action. However, no study so far has shown the anti-nociceptive effect of isotalatizidine in a neuropathic pain model. We observed that isotalatizidine not only relieved acetic acid-induced somatic pain in a mouse model but also alleviated mechanical allodynia in the ipsilateral paw of a CCI-induced neuropathic pain model in a dose-dependent manner, without affecting the normal nociceptive response in the contralateral paw.

The activation of spinal microglia plays an important role in initiating and sustaining chronic neuropathic pain, with the intervention of the MAPK signaling pathway. Activated MAPKs induce diverse intracellular response and are also involved in maintaining hypersensitivity in neuropathic pain via transcriptional and non-transcriptional regulation of downstream factors [21, 34]. Consistent with this, MAPKs and microglial activation inhibitors effectively attenuate neuropathic pain in different models [35]. We found that isotalatizidine stimulated p38 and ERK1/2 in cultured BV-2 cell line or primary microglia, which was completely inhibited by the respective inhibitors. Our findings are consistent with that of Huang et al. who demonstrated that cynandione A, a C_19_-diester and monoester diterpenoid alkaloid structurally similar to isotalatizidine and extracted from *Cynanchi Wilfordii* Radix, exerted its anti-nociceptive effect by non-selectively activating MAPKs [36]. We hypothesized therefore that the activation of p38 or ERK1/2 mediated the analgesic effect of iaotalatizidine in the neuropathic pain model.

As a downstream target of MAPK, CREB plays a pivotal role in the development of neuropathic pain by regulating transcription and secretion of diverse neurotransmitters. Binding of cAMP to the regulatory subunit of protein kinase A (PKA) phosphorylates CREB, which eventually regulates multiple cellular events. Previous studies have shown that activated p38 or ERK1/2 induces CREB phosphorylation in microglial or neuronal cells [37, 38]. Consistent with this, isotalatizidine increased the levels of p-CREB in microglial cells, which was blocked by inhibiting ERK1/2 but not p38 or JNK. This suggested that isotalatizidine-induced phosphorylation of CREB was specifically mediated by the ERK1/2 pathway.

**Fig. 6:**
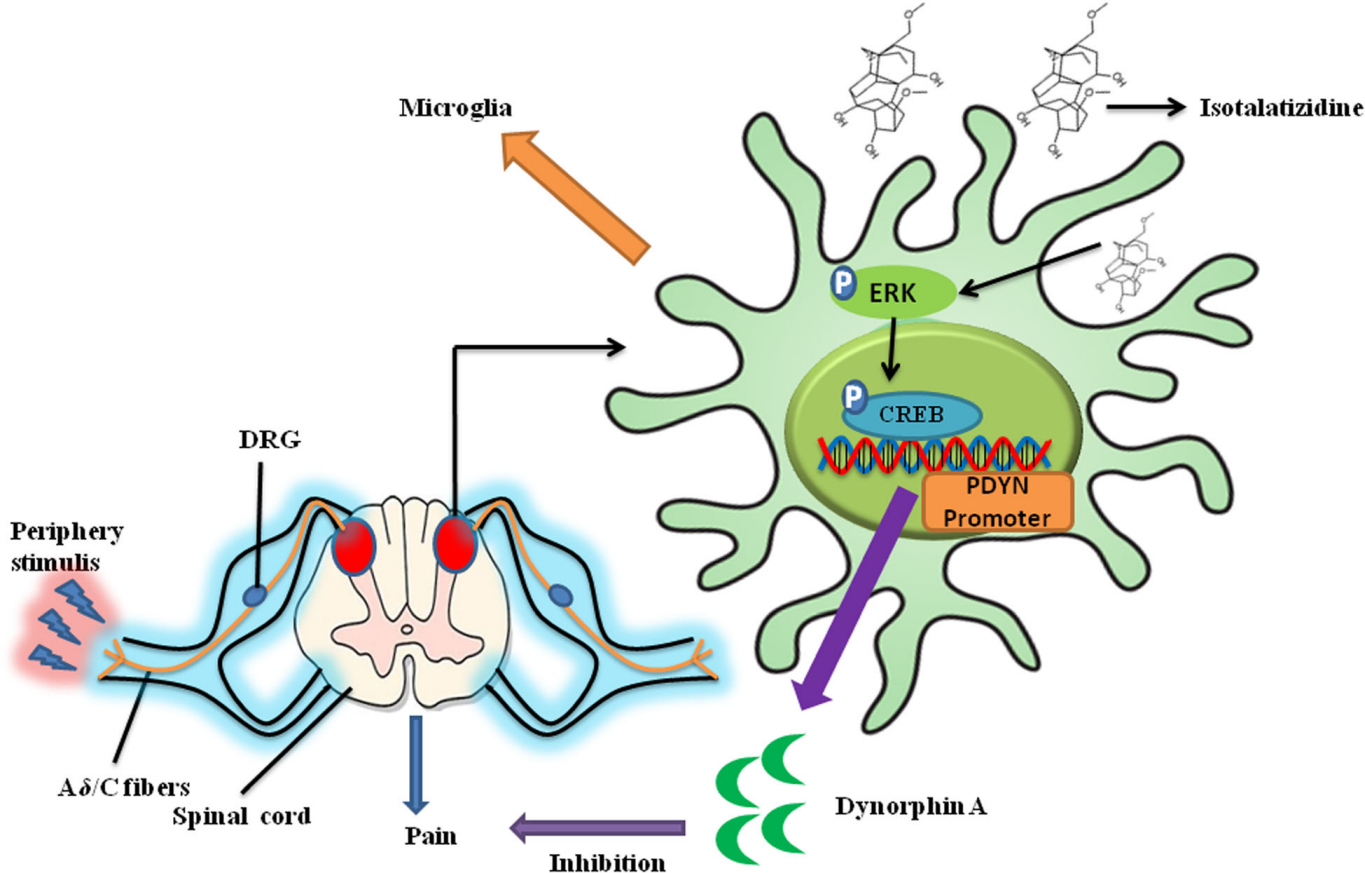
Proposed analgesic mechanism of isotalatizidine for ERK/CREB signal pathway-induced expression of dynorphin A in spinal microglia

As an endogenous ligand, dynorphin A is closely related to neuropathic pain by binding to the κ-opioid receptor, but the detailed antinociceptive mechanism of dynorphin A is still unclear [39, 40]. Elevated dynorphin A has previously been implicated in the development and maintenance of neuropathic pain, since intrathecal administration of anti-dynorphin A serum ameliorated pain in an animal model [41]. However, several studies showed that exogenous agents could induce the production of dynorphin A by microglia or neurons leading to an analgesic effect in CCI-induced model [42–44]. Further investigating indicated that the analgesic effect by dynorphin A failed to administrate specific anti-dynorphin A antibody or selective κ-opioid receptor antagonist GNTI. In the present study, we also found that isotalatizidine induced the secretion of dynorphin A in the spinal cord tissue in CCI-induced neuropathic pain model, and this elevated dynorphin A assuages rather than aggravates neuropathic pain. Furthermore, we found that the isotalatizidine-induced secretion of dynorphin A could be blocked by the selective ERK1/2 inhibitor or selective CREB inhibitor, but not p38 inhibitor. Further experimental also supported the above results, which indicated that isotalatizidine induced a mRNA expression of prodynorphin in cultured microglia of BV-2, and this increased expression also could be prevented by ERK1/2 inhibitor or CREB inhibitor, respectively. Taken together, the anti-nociception action of isotalatizidine in CCI-induced neuropathic pain model is mediated via the activation of the ERK1/2-CREB-dynorphin A axis. The proposed signal transduction pathway is presented in Fig. 6. Our findings provide novel insights into the pathological role of microglial activation in neuropathic pain and the underlying mechanisms.

## Conclusion

Isotalatizidine alleviated mechanical allodynia in neuropathic pain in a dose-dependent manner by activating the ERK1/2 pathway and phosphorylating CREB, which triggered dynorphin A release from the microglia. Our findings provided primary pharmacological evidence for the potential use of isotalatizidine against neuropathic pain.

## Data Availability

The datasets during and/or analyzed during the current study are available from the corresponding author on reasonable request.

## Abbreviations

CCI: Chronic constrictive injury
CNS: Central nervous system
CREB: cAMP-response element binding protein
ERK: Extracellular signal-regulated kinase
JNK: c-Jun N-terminal kinase
MAPK: Mitogen-activated protein kinase
PBS: Phosphate-buffered saline
